# A 3-year cohort study of the natural history of spinocerebellar ataxia type 6 in Japan

**DOI:** 10.1186/s13023-014-0118-4

**Published:** 2014-07-23

**Authors:** Kenichi Yasui, Ichiro Yabe, Kunihiro Yoshida, Kazuaki Kanai, Kimihito Arai, Mizuki Ito, Osamu Onodera, Shigeru Koyano, Eiji Isozaki, Setsu Sawai, Yoshiki Adachi, Hidenao Sasaki, Satoshi Kuwabara, Takamichi Hattori, Gen Sobue, Hidehiro Mizusawa, Shoji Tsuji, Masatoyo Nishizawa, Kenji Nakashima

**Affiliations:** 1 Division of Neurology, Department of Brain and Neurosciences, Faculty of Medicine, Tottori University, 36-1 Nishi-cho, Yonago 683-8504, Japan; 2 Department of Neurology, Graduate School of Medicine, Hokkaido University, Sapporo, Japan; 3 Divisions of Neurogenetics, Department of Brain Disease Research, Shinshu University School of Medicine, Matsumoto, Japan; 4 Department of Neurology, Graduate School of Medicine, Chiba University, Chiba, Japan; 5 Department of Neurology, National Hospital Organization Chiba-East-Hospital, Chiba, Japan; 6 Department of Neurology, Graduate School of Medicine, Nagoya University, Nagoya, Japan; 7 Department of Molecular Neuroscience, Resource Branch for Brain Disease Research, Brain Research Institute, Niigata University, Niigata, Japan; 8 Department of Neurology and Stroke Medicine, Graduate School of Medicine, Yokohama City University, Yokohama, Japan; 9 Department of Neurology, Tokyo Metropolitan Neurological Hospital, Tokyo, Japan; 10 Department of Neurology, National Hospital Organization Matsue-Hospital, Matsue, Japan; 11 Department of Neurology and Neurological Science, Graduate School of Medical and Dental Science, Tokyo Medical and Dental University, Tokyo, Japan; 12 National Center Hospital, National Center of Neurology and Psychiatry, Tokyo, Japan; 13 Department of Neurology, Graduate School of Medicine, The University of Tokyo, Tokyo, Japan; 14 Department of Neurology, Clinical Neuroscience Branch, Brain Research Institute, Niigata University, Niigata, Japan

**Keywords:** Barthel Index, CAG repeat, International Cooperative Ataxia Rating Scale, Intractable diseases research, Scale for the Assessment and Rating of Ataxia, Spinocerebellar ataxia

## Abstract

**Background:**

Only a few prospective studies have determined which clinical symptoms and factors are associated with the disease severity of spinocerebellar ataxia type 6 (SCA6). A multicenter longitudinal cohort study was conducted to clarify both the natural history of SCA6 in Japan and the factors influencing disease progression.

**Methods:**

Patients were consecutively recruited between 2007 and 2008. Scores from the Scale for the Assessment and Rating of Ataxia (SARA) and Barthel Index (BI) were collected prospectively each year. Additionally, data from the Japan intractable diseases research (IDR) registry were collected both retrospectively, from 2003 to 2006, and prospectively, from 2007 to 2010. As a result, we were able to collect 3 years of retrospective data and 4 years of prospective data during the course of 3 yearly visits.

**Results:**

Forty-six patients were registered. The follow-up rate of the third year was 93%. The SARA scores worsened significantly each year. Over 3 years, the decline of the SARA scores was 1.33 ± 1.40 points/year. The results of multivariate analysis of the decline of the SARA score were not significant. The IDR scores correlated well with the SARA and BI scores. Kaplan-Meier curves of 7 years of data from the IDR registry illustrated the correlation between the ability to walk and the time course of the disease.

**Conclusions:**

Information regarding the progression of ataxia and the decline in the activities of daily living (ADL) in patients with SCA6 was obtained by a 3-year cohort study and a 7-year IDR study. The decline of the SARA score of patients with SCA6 was 1.33 ± 1.40 points/year. The results elucidate the natural history of SCA6, factors influencing disease severity, and utility of data from the IDR registry of Japan.

## Background

The spinocerebellar ataxias (SCAs) are neurodegenerative diseases characterized by oculomotor disturbances, dysarthria, limb and truncal ataxia, gait disturbances, and additional variable symptoms [1]. In the 1990’s, genetic mapping studies in patients with autosomal dominant cerebellar ataxias (ADCAs) identified 7 polyglutamine diseases: SCA type 1 [SCA1], SCA2, SCA3, SCA6, SCA7, SCA17, and dentatorubral-pallidoluysian atrophy [DRPLA] [2].

Although numerous studies have described the clinical manifestations of the SCAs [2]-[4], very few prospective studies have examined which clinical symptoms and factors are associated with disease severity. Knowledge of the natural history of the SCAs is required to counsel patients and to design interventional trials. One cohort study of SCA, the European EUROSCA natural history study, was a multicenter longitudinal study that included 526 patients with SCA1, SCA2, SCA3, and SCA6 [5]-[8]. A 2-year follow-up study of EUROSCA used several scales, including the Scale for the Assessment and Rating of Ataxia (SARA), to describe disease progression and identify factors that specifically affected this process [8]. Recently, Ashizawa et al. reported the results of a prospective study of the natural history of the SCAs in the United States [9]. We designed a multicenter longitudinal cohort study of the natural history of SCA6 in Japan. Our study used the SARA and the Barthel Index (BI). SCA6 was selected because of the high prevalence of patients with this disease in Japan.

In Japan, the Ministry of Health, Labour, and Welfare has established a national registry system for the survey of ‘intractable diseases’ such as the SCAs [10],[11]. The Ministry is conducting a project that subsidizes the medical expenses of patients with an ‘intractable disease’. This project also supports research activities on these diseases because effective treatments have not yet been established and the affected patients have considerable disabilities. Each year, patients with an ‘intractable disease’ submit a ‘clinical inquiry sheet,’ which is completed by their physician, to the IDR. Since 2003, the inquiry sheet for patients with an SCA has included 5 items from the International Cooperative Ataxia Rating Scale (ICARS) and 6 items from the BI [12],[13]. The purposes of the IDR registry are to provide financial support for patients and investigate the patients’ clinical states; however, data from the present IDR registry have not yet been utilized for longitudinal natural history studies.

The primary aims of this study were to longitudinally and quantitatively investigate the clinical severity, disease progression, and natural history of SCA6, to identify factors that specifically affected disease progression in a 3-year prospective study in Japan, and to compare the results of this study with those of previous studies. The secondary aim was to examine the reliability and utility of the data collected by the Ministry of Health, Labour, and Welfare’s national IDR registry.

## Methods

### Patient registration

The present study was performed at 8 centers (Hokkaido University, Niigata University, Chiba University, National Hospital Organization Chiba-East-Hospital, Tokyo Metropolitan Neurological Hospital, Shinsyu University, Nagoya University, and Tottori University) belonging to the Research Committee for Ataxic Disease. This committee is part of the Ministry of Health, Labour, and Welfare of Japan. The written informed consent form, which was approved by the institutional review board of all centers and by the Ethics Committee of the Tottori University Hospital, was signed by all study participants. Patients with SCA6 who were being treated at any of the 8 centers were consecutively recruited between 2007 and 2008. A linkable, anonymizing registration system was used to register all patients.

The patients did not provide DNA samples for this study and therefore diagnoses based on DNA analyses were made in accordance with the protocols being used at each center. Information regarding the CAG repeat length of the expanded allele of the alpha 1A P/Q type voltage-dependent calcium channel gene (*CACNA1A*) of each patient was obtained from the medical records of the respective center.

### Procedures of the 3-year prospective study

Registration and follow-up evaluations were performed from April to July during the annual registration period of the Japan IDR registry; therefore, follow-up investigations were performed at the same time each year. As part of the prospective study, all patients were assessed with the SARA and the BI each year. The SARA was used to assess the degree of ataxia. The SARA consists of 8 items: gait, stance, sitting, speech, finger chase, nose-to-finger test, fast alternating hand movements, and heel-to-shin slide. Each item has its own subscore. The SARA grades ataxia on a scale of 0 to 40, with 0 indicating the absence of ataxia and 40 indicating the most severe degree of ataxia. The Japanese SARA is well validated and can be administered quickly [14],[15]. The BI was used to assess how well a patient performs the activities of daily living (ADL). The BI grades activity on a scale of 0 to 100, with 0 indicating that a person cannot care for him/herself and 100 indicating that a person can care for him/herself. All investigators were board-certified neurologists and were experienced in the use of the applied scales.

### Procedures of the 7-year IDR registry study

The IDR inquiry sheet for patients with an SCA includes 5 items from the ICARS (walking, standing with eyes open, body sway with feet together and eyes closed, knee-tibia test of the worse foot, and finger-to-nose test of the worse hand [decomposition and dysmetria]) and 6 items from the BI (feeding, bathing, grooming, dressing, mobility, and stairs) [11]-[13]. The total number of points obtained from the IDR inquiry sheet for the 5 items from the ICARS and the 6 items from the BI was referred to as the IDR-ICARS and IDR-BI scores, respectively. The IDR-ICARS grades ataxia on a scale of 0 to 26, with 0 indicating the least impaired condition. The IDR-BI grades activity on a scale of 0 to 55, with 0 indicating the most impaired condition. The 5 items of the IDR-ICARS were selected because these subscores correlated well with disease duration in Japanese patients with cerebellar ataxia [10].

The IDR inquiry sheet was last modified in 2003. For the retrospective portion of the IDR registry study, we collected the IDR inquiry sheets from 2003 onwards. The inquiry sheets were collected from the patients’ medical records, starting in the year they registered for the study. IDR data collection continued prospectively each year. The utility of the IDR registry was evaluated by analyzing a total of 7 years’ worth of data.

### Data analysis

The differences between the scores obtained at registration and those obtained at each year’s evaluation were referred to as the ∆ scores, and the differences between the scores obtained at registration and the scores obtained at the last evaluation were referred to as the total ∆ scores. The total ∆/year was calculated by dividing the total ∆ score by the number of follow-up years.

Statistical analyses were performed with IBM SPSS Statistics software version 19 (SPSS Inc., Chicago, IL). The test results were considered significant at the .05 level. The Mann–Whitney test was used to compare the clinical characteristics of the male and female patients. Correlations between clinical scores and covariates were tested by using the Pearson correlation test. Data for disease progression were analyzed by using the Friedman test followed by post hoc Wilcoxon signed rank tests. The rate at which patients became wheelchair dependent was calculated from the data of the 7-year IDR registry study by using the Kaplan-Meier method. The CAG repeat length of the normal *CACNA1A* allele of each patient could not be collected in this study; therefore, we analyzed the repeat lengths of the expanded alleles, with the exception of the repeat lengths of the 3 patients who are homozygous for the expanded allele. For the cross-sectional study, an analysis of covariance at registration was performed with the SARA score as the dependent variable and sex, age at onset, disease duration, and CAG repeat length of the expanded *CACNA1A* allele as independent variables. For the prospective study, an analysis of covariance was performed with the total ∆SARA/year as the dependent variable and sex, age at onset, disease duration, CAG repeat length of the expanded *CACNA1A* allele, and SARA score at registration as independent variables. Age at registration was not included in the model, as age at registration was recorded as the sum of age at onset and disease duration. The test results were considered significant at the .01 level for the multivariate analysis.

## Results

### Patient characteristics

The study population consisted of 46 patients with SCA6 who belonged to 44 families. The SARA and IDR-ICARS scores of female patients were significantly lower than those of male patients. Although the age at onset, age at registration, and disease duration tended to be lower in female patients than in male patients, the differences were not statistically significant. Similarly, although the BI and IDR-BI scores of female patients tended to be higher than those of male patients, the differences were not statistically significant (Table 1).

**Table 1:** Demographic, genetic, and clinical characteristics of the study population

	**All patients**	**Male patients**	**Female patients**
No.	46	23	23
Age at onset, y	48.0 ± 9.3 (31–66)	48.8 ± 10.0	47.2 ± 8.6
Age at registration, y	63.0 ± 9.6 (41–78)	64.4 ± 10.0	61.5 ± 9.2
Disease duration, y	15.0 ± 8.0 (3–40)	15.6 ± 7.5	14.3 ± 8.7
SARA score, points (Range, 0–40)	15.9 ± 7.1 (4–33)	18.2 ± 6.2	13.6 ± 7.3*
BI score, points (Range, 0–100)	77.4 ± 22.4 (15–100)	72.2 ± 23.0	82.6 ± 20.9
IDR-ICARS score, points (Range, 0–26)	14.8 ± 6.0 (5–26)	16.6 ± 5.6	13.1 ± 6.1*
IDR-BI score, points (Range, 0–55)	36.7 ± 15.1 (5–55)	33.5 ± 16.1	40.0 ± 13.7
CAG repeat length of the expanded *CACNA1A* allele^a^	23.2 ± 1.4 (21–27)	23.2 ± 1.3	23.3 ± 1.6

Where applicable, the values are given as the mean ± standard deviation (range).

Abbreviations: BI = Barthel Index; *CACNA1A* = alpha 1A P/Q type voltage-dependent calcium channel gene; CAG = cytosine-adenine-guanine; ICARS = International Cooperative Ataxia Rating Scale; IDR = Intractable Diseases Research; SARA = Scale for the Assessment and Rating of Ataxia.

IDR-BI = total points of 6 items from the BI assessed by the IDR registry.

IDR-ICARS = total points of 5 items from the ICARS assessed by the IDR registry.

^a^Three patients are homozygous for the expanded allele (repeat lengths: 20, 22, and 24). The mean ± standard deviation was applied for 43 patients heterozygous for the expanded allele (22 male and 21 female patients).

*The scores of female patients were significantly lower than those of male patients (*P* < .05, Mann–Whitney test).

### Correlations between clinical scores and factors at registration

A patient’s age at the time of registration and the duration of his/her disease were correlated with clinical scores; however, a patient’s age at the time of disease onset and the CAG repeat length of the expanded *CACNA1A* allele were not correlated with those scores (Table 2).

**Table 2:** Correlations between the SARA, IDR-ICARS, BI, and IDR-BI scores and patients’ demographics

	**SARA**	**IDR-ICARS**	**BI**	**IDR-BI**
Age at onset	NS	NS	NS	NS
Age at registration	0.36^b^	0.43^c^	−0.45^c^	−0.57^c^
Disease duration	0.35^b^	0.45^c^	−0.35^b^	−0.44^c^
CAG repeat length of the expanded *CACNA1A* allele^a^	NS	NS	NS	NS

Correlation coefficients are presented.

Abbreviations: BI = Barthel Index; *CACNA1A* = the alpha 1A P/Q type voltage-dependent calcium channel gene; CAG = cytosine-adenine-guanine; ICARS = International Cooperative Ataxia Rating Scale; IDR = Intractable Diseases Research; NS = not significant; SARA = Scale for the Assessment and Rating of Ataxia.

IDR-BI = total points of 6 items from the BI assessed by the IDR registry.

IDR-ICARS = total points of 5 items from the ICARS assessed by the IDR registry.

^a^Data on the CAG repeat length were analyzed for 43 patients heterozygous for the expanded allele.

^b^*P* < .05 (statistical analysis conducted with the Pearson correlation test).

^c^*P* < .01 (statistical analysis conducted with the Pearson correlation test).

**Figure 1:**
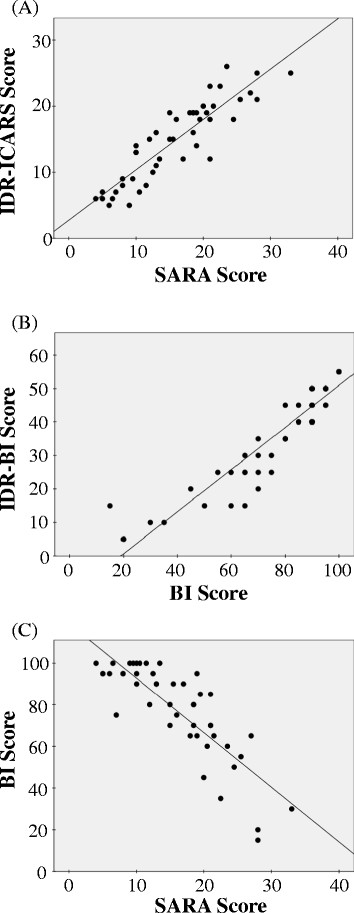
**Relationships between the clinical scales used by the prospective study and by the IDR registry. (A)** The relationship between the IDR-ICARS and SARA scales. The IDR-ICARS scores correlated well with the SARA scores (R = 0.892, *P* < .001, Pearson correlation test). **(B)** The relationship between the IDR-BI and BI scales. The IDR-BI scores correlated well with the BI scores (R = 0.928, *P* < .001, Pearson correlation test). **(C)** The relationship between the SARA and BI scales. The BI scores were inversely correlated with the SARA scores (R = −0.828, *P* < .001, Pearson correlation test). Abbreviations: BI = Barthel Index; ICARS = International Cooperative Ataxia Rating Scale; IDR = Intractable Diseases Research; SARA = Scale for the Assessment and Rating of Ataxia.

The patients’ IDR-ICARS and IDR-BI scores correlated well with their SARA and BI scores, respectively (Figure 1A, B). The correlation coefficients of the SARA and IDR-ICARS scores and the BI and IDR-BI scores were 0.89 and 0.93, respectively (*P* < .001). The BI scores were inversely correlated with the SARA scores (R = −0.83, *P* < .001). Patients with a SARA score of less than 10 points maintained a high BI score. Conversely, among patients with a SARA score of more than 10 points, those with a higher SARA score had a lower BI score (Figure 1C).

The results of multivariate analysis of the patients’ SARA scores at the time of registration are presented in Table 3. An analysis of covariance with the SARA score as the dependent variable and clinical factors as independent variables produced multivariate models that explained 39.5% of the variance of the SARA scores. The SARA scores were influenced by sex, age at onset, disease duration, and CAG repeat length of the expanded *CACNA1A* allele.

**Table 3:** Results of multivariate analysis for the SARA score at registration

***R***^***2***^	***P***	***Effect***	***Estimate***	***SE***	***β***	***t***	***P***
0.395	.001						
		Intercept	−45.558	21.830	n/a	−2.087	.044
		Sex	−5.669	1.775	−.415	−3.194	.003
		Age at onset	.338	.117	.451	2.877	.007
		Disease duration	.252	.122	.285	2.067	.046
		CAG repeat length of the expanded *CACNA1A* allele	2.132	.812	.421	2.627	.012

R^2^ = Coefficient of determination; β = standard coefficient.

Abbreviations: *CACNA1A* = alpha 1A P/Q type voltage-dependent calcium channel gene; CAG = cytosine-adenine-guanine; n/a = not applicable; SARA = Scale for the Assessment and Rating of Ataxia; SE = standard error.

### Findings of the 3-year prospective study

The data obtained from the prospective study were used to obtain information on disease progression. This information is presented in Table 4 (5 right most columns). During the 3-year follow-up period, 2 patients died and 1 patient dropped out. The causes of the 2 deaths were suffocation and aplastic anemia. One untraceable patient could not continue to visit because of his disability. The follow-up rate of the third year was 93%. The total number of evaluations for the prospective study was 177. The SARA scores worsened significantly each year. The ∆SARA/year at the 1-, 2-, and 3-year follow-up evaluations was 1.35 ± 1.70 (mean ± SD), 1.39 ± 1.39, and 1.24 ± 1.06 points/year, respectively. The total ∆SARA/year was 1.33 ± 1.40 points/year. The ∆SARA/year was 1.08 ± 0.92 points/year for male patients and 1.56 ± 1.71 points/year for female patients. The difference of the ∆SARA/year between genders was not significant. Each year, the SARA scores of patients who scored between 0 and 24.5 points changed by similar amounts (∆SARA/year = 1.48 ± 1.86 points/y), but the amount of change was smaller for patients with scores of more than 25 points (∆SARA/year = 0.48 ± 1.42 points/y).

**Table 4:** Time course of disease progression based on the SARA, BI, IDR-ICARS and IDR-BI scores

**Follow-up year**	**−4**	**−3**	**−2**	**−1**	**Registration**	**1**	**2**	**3**	**Total**
N (follow-up rate%)	29	35	38	40	46(100)	44(96)	44(96)	43(93)	177*
Death, n					0	1	0	1	2
Withdrawal, n					0	1	0	0	1
SARA score, points					15.9 ± 7.1	16.8 ± 6.9^a^	18.2 ± 7.2^a^	19.1 ± 6.9^a^	17.5 ± 7.1
ΔSARA, points					0	1.35 ± 1.70	2.78 ± 2.78	3.73 ± 3.17	n/a
ΔSARA/year, points/y					0	1.35 ± 1.70	1.39 ± 1.39	1.24 ± 1.06	1.33 ± 1.40
BI score, points					77.4 ± 22.4	77.4 ± 22.3^b^	73.8 ± 23.7^b^	75.0 ± 23.2	75.9 ± 22.8
IDR-ICARS score, points	13.9 ± 5.9	13.7 ± 5.9	14.1 ± 6.0^c^	14.6 ± 5.7	14.8 ± 6.0	15.0 ± 5.4^b^	16.0 ± 5.7^b^	16.2 ± 5.6	14.9 ± 5.8
ΔIDR-ICARS, points	−2.28 ± 3.59	−2.06 ± 3.27	−1.29 ± 2.98	−0.50 ± 2.32	0	0.59 ± 1.65	1.59 ± 2.34	1.77 ± 2.79	n/a
ΔIDR-ICARS/year, points/y	−0.57 ± 0.90	−0.69 ± 1.09	−0.64 ± 1.49	−0.50 ± 2.32	0	0.59 ± 1.65	0.80 ± 1.17	0.59 ± 0.93	0.63 ± 1.45^d^
IDR-BI score, points	37.1 ± 14.0	38.6 ± 14.2	38.4 ± 13.8^c^	37.4 ± 14.1	36.7 ± 15.1	37.6 ± 15.0	34.9 ± 14.8^b^	35.8 ± 14.9	36.3 ± 14.9

Where applicable, the values are given as the mean ± standard deviation.

Comparisons of the IDR-ICARS, IDR-BI, SARA, and BI scores of each year were made by using the Friedman test followed by the Wilcoxon signed rank test as a post hoc test.

Abbreviations: BI = Barthel Index; ICARS = International Cooperative Ataxia Rating Scale; IDR = Intractable Diseases Research; n/a = not applicable; SARA = Scale for the Assessment and Rating of Ataxia.

IDR-BI = total points of 6 items from the BI assessed by the IDR registry.

IDR-ICARS = total points of 5 items from the ICARS assessed by the IDR registry.

ΔIDR-ICARS and ΔSARA = differences between the scores obtained at the follow-up evaluations and those obtained at registration for the IDR-ICARS and SARA, respectively.

^a^*P* < .001, versus the previous year for the prospective study.

^b^*P* < .05 versus the previous year for the prospective study.

^c^*P* < .05 versus the next year for the retrospective study.

^d^Total of the IDR-ICARS/year calculated with the absolute value of each ΔIDR-ICARS/year excluding that of the registration year.

*Total number of evaluations conducted during the prospective arm of the study.

Among the subscores of the SARA scale, the subscores for gait (∆/year = 0.27 points/y), stance (∆/year = 0.22 points/y), and fast alternating hand movements (∆/year = 0.24 points/y) were worse than the 5 other subscores (sitting: ∆/year = 0.10 points/y, speech: ∆/year = 0.16 points/y, finger chase: ∆/year = 0.06 points/y, nose-to-finger test: ∆/year = 0.10 points/y, heel-to-shin slide: ∆/year = 0.10 points/y) at the third year. The results of multivariate analysis for the decline in the SARA score were not significant.

### Findings of the 7-year IDR registry study

The data from the 7-year IDR registry study were used to analyze disease progression. The results of this analysis are presented in Table 4 (last 4 rows). The ∆IDR-ICARS/year of the prospective portion of the study (absolute value 0.66 ± 1.28 points/y) was similar to that of the retrospective portion (absolute value 0.60 ± 1.59 points/y). The IDR-ICARS scores changed linearly in the retrospective and prospective portions of the study. The regression coefficient of the linear regression analysis was 0.63. Among the subscores of the IDR-ICARS scale, the subscore for walking (∆/year = 0.23 points/y) was worse than the 4 other subscores (standing: ∆/year = 0.12 points/y, body sway: ∆/year = 0.11 points/y, knee-tibia test: ∆/year = 0.05 points/y, and finger-to-nose test: ∆/year = 0.07 points/y) at the third year.

**Figure 2:**
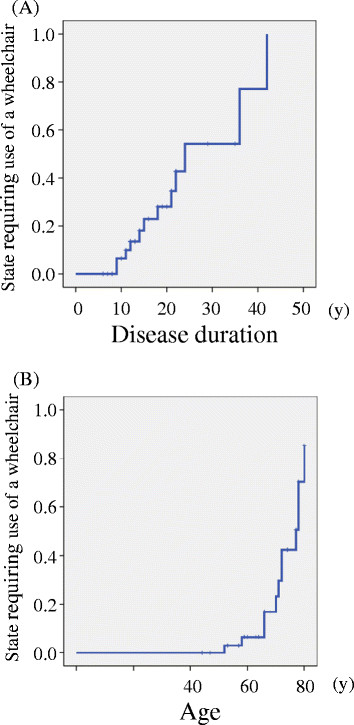
**Utilization of ADL states as milestones indicating disease progression.** Data from the 7-year IDR registry study was used to obtain information regarding the time that elapsed before patients became wheelchair dependent (**A**: Disease duration, **B**: Patient’s age). Among 37 patients with SCA6, 12 became wheelchair dependent during the 7 years of the study. The Kaplan-Meier curves are shown. The medians (95% confidence interval) of disease duration and age for those patients who became wheelchair dependent were 24.0 years (12.8 – 35.2 y) and 77.0 years (71.6 – 82.4 y), respectively. Abbreviations: ADL = activities of daily living; SARA = Scale for the Assessment and Rating of Ataxia; SCA6 = spinocerebellar ataxia type 6.

Among the 46 patients with SCA6 who participated in this study, 9 already used a wheelchair when they filed their first IDR inquiry sheet. During the 7-year study period of the IDR registry, 12 of the remaining 37 patients became wheelchair dependent. Figure 2 panels A and B show the rates at which patients became wheelchair dependent. These rates are based on disease duration and age, respectively. All patients with a disease duration of less than 8 years and patients who were younger than 51 years could walk during the entire study. During the course of the study, patients with a disease duration of more than 9 years and patients who were older than 52 years gradually became wheelchair dependent. The medians of disease duration and age in patients who needed to use a wheelchair were 24.0 years (95% confidence interval [95% CI], 12.8 - 35.2 y) and 77.0 years (95% CI, 71.6 - 82.4 y), respectively. The 9 patients who were wheelchair dependent when they filed their first IDR inquiry sheet had a disease duration of more than 11 years and an IDR-ICARS score of more than 17 points, and all were older than 56 years.

## Discussion

This 3-year prospective study elucidated the quantitative natural history of SCA6 in Japan. Although several previous studies have used a cross-sectional or retrospective design to describe the clinical characteristics of SCA6 [16]-[18], ours is the first to use SARA and BI scores to prospectively assess a Japanese cohort. Furthermore, this study provides an accurate natural history of SCA6 because of the high follow-up rate. Our success was possible because the study was performed in 8 centers belonging to the Research Committee for Ataxic Disease. This committee is part of the Ministry of Health, Labour, and Welfare of Japan. In addition, the follow-up investigations were performed at the same time each year, during the annual registration period of the Japan IDR registry.

Regrettably, one disadvantage of this study is that DNA was not collected: only data for the CAG repeat length of the expanded allele of the *CACNA1A* gene was used in our analysis. We did not analyze the repeat lengths of the patients who are homozygous for the expanded *CACNA1A* allele. These data were collected from the patients’ medical records. Given that the genetic data of this study were partially limited, we emphasized the clinical courses of the patients.

The correlation analysis of the patients’ clinical scores and clinical factors revealed that a patient’s age at the time of registration and the duration of his/her disease were correlated with the SARA score (Table 2). These results and the corresponding correlation coefficients are similar to those of previous reports [6]. Furthermore, the results of this study clarified the correlation between the SARA and BI scores (Figure 1C). Among the 46 participants in this study, female patients tended to be younger and have a shorter disease duration than male patients; however, the differences were not statistically significant. On the other hand, the SARA and IDR-ICARS scores of female patients were significantly less than those of male patients. Thus, the patients’ clinical backgrounds differed slightly between genders.

During the 3 years of this study, the SARA scores declined by 1.33 points/year, and the ∆SARA/year did not change significantly. This decline in the SARA score is greater than the 0.35 points/year decline that was observed for the first year of the EUROSCA study but it is similar to that observed for the second year (1.44 points/y) [8]. In a study conducted in the United States, the SARA score of patients with SCA6 declined by 0.87 ± 0.28 points/year [9]. In another study conducted in Asia, the SARA score of patients with SCA6 declined by 2.04 ± 0.76 points/year [19]. Although the reason for these differences is unclear, several explanations are possible. First, the backgrounds of the patients may be an important factor. Second, the dependence of the ∆SARA on the SARA score may be another reason why the scores differ: the ∆SARA/year for the patients with SARA scores of 25 points or more was less than that for the patients with SARA scores of less than 25 points. This finding may have important implications for the design of future clinical trials. Judging from the results of this study, we conclude that it may be appropriate to exclude patients with SARA scores of greater than 25 points. Third, the follow-up rate and the number of patients enrolled may also have been associated with the differences in the results of these studies.

The results of multivariate analysis for the decline in the SARA scores were not significant. The findings of a 2-year follow-up study by Jacobi et al. indicate that disease duration and CAG repeat length of the normal *CACNA1A* allele were independent factors associated with the decline in the SARA score in male patients with SCA6 [8]. We currently have no explanation for Jacobi et al’s study because the genetic data and number of patients of this study were limited. Disease progression in patients with SCA6 may be affected by various factors including disease duration, age, and disease severity at the time of registration as well as by the CAG repeat length of the *CACNA1A* alleles. The 3-year prospective observation period of our study is longer than those of previous studies, and the length of the observation period is one of the most important factors for a study of the natural history of a disease. Furthermore, as the number of follow-up years increases, disease progression may become increasingly homogenous.

In addition to data generated by the 3-year prospective study, the 7-year IDR registry study provided long-term information on the natural history of SCA6. The amount of change of the IDR scores was small; therefore, the IDR inquiry sheet may not be suitable for use in clinical trials. However, the IDR inquiry sheet is simple and it can be completed in the setting of a typical daily clinic. Moreover, the IDR-ICARS and IDR-BI scores correlated well with the SARA and BI scores, respectively. Therefore, the IDR inquiry sheet could be useful for an uncomplicated follow-up study. The 7-year IDR registry study allowed us to monitor the long-term progression of ataxia, as indicated by the changes in the IDR-ICARS scores and the decline in the ADL, which are measured by the IDR-BI. Kaplan-Meier curves described the correlation between the ability to walk and the time course of the disease. A long-term period of observation is indispensable for a thorough understanding of the natural history of the SCAs.

## Conclusions

This study clarified the natural history of SCA6 by performing a 3-year prospective study and by analyzing 7 years’ worth of data from the IDR registry. The decline of the SARA score of patients with SCA6 was 1.33 ± 1.40 points/year. Additionally, our study demonstrated both the limitations and the advantages of Japan’s IDR registry. Finally, the results of this study may have important implications for the planning of future clinical trials that investigate new treatments for the SCAs.

## Abbreviations

ADCA: Autosomal dominant cerebellar ataxia

ADL: Activities of daily living

BI: Barthel index

*CACNA1A*: Alpha 1A P/Q type voltage-dependent calcium channel gene

CAG: Cytosine-adenine-guanine

ICARS: International Cooperative Ataxia Rating Scale

IDR: Intractable diseases research

SARA: Scale for the Assessment and Rating of Ataxia

SCA: Spinocerebellar ataxia

## Competing interests

The authors declare that they have no competing interests.

## Authors’ contributions

KY: research project execution, statistical analysis design, statistical analysis of data, drafting the manuscript for intellectual content; IY: research project execution, manuscript review and critique; KY: research project execution, manuscript review and critique; KK: research project execution, statistical analysis and critique, manuscript review and critique; KA: research project execution, manuscript review and critique; MI: research project execution, manuscript review and critique; OO: research project execution, manuscript review and critique; SK: research project execution, manuscript review and critique; EI: research project execution, manuscript review and critique; SS: research project execution; YA: research project execution; HS: research project organization, research project execution, manuscript review and critique, study supervision; SK: research project execution, manuscript review and critique; TH: research project execution, manuscript review and critique; GS: research project organization, research project execution, manuscript review and critique; HM: research project organization, manuscript review and critique; ST: research project organization, manuscript review and critique; MN: research project organization, research project execution, manuscript review and critique, study supervision; KN: research project organization, research project execution, statistical analysis design, statistical analysis and critique, manuscript review and critique, study supervision. All authors read and approved the final manuscript.
